# Microfluidic-Assisted Synthesis of Metal—Organic Framework —Alginate Micro-Particles for Sustained Drug Delivery

**DOI:** 10.3390/bios13070737

**Published:** 2023-07-17

**Authors:** Akhilesh Bendre, Vinayak Hegde, Kanalli V. Ajeya, Subrahmanya Thagare Manjunatha, Derangula Somasekhara, Varalakshmi K. Nadumane, Krishna Kant, Ho-Young Jung, Wei-Song Hung, Mahaveer D. Kurkuri

**Affiliations:** 1 Centre for Research in Functional Materials (CRFM), JAIN (Deemed-to-be University), Jain Global Campus, Bengaluru 562112, Karnataka, India; akhilesh.bendre@jainuniversity.ac.in (A.B.); vinayakhegde2020@gmail.com (V.H.); 2 Department of Environment and Energy Engineering, Chonnam National University, 77 Yongbong-ro, Buk-gu, Gwangju 61186, Republic of Korea; ajeyhegde94@gmail.com; 3 Advanced Membrane Materials Research Center, Graduate Institute of Applied Science and Technology, National Taiwan University of Science and Technology, Taipei 10607, Taiwan; mssubrahmanya@gmail.com; 4 Department of Biotechnology, JAIN (Deemed-to-be-University), School of Sciences, JC Road, 34, 1st Cross Road, Sudharna Nagar, Bengaluru 560027, Karnataka, India; d.somasekhara@jainuniversity.ac.in (D.S.); kn.varalakshmi@jainuniversity.ac.in (V.K.N.); 5 Biomedical Research Center (CINBIO), University of Vigo, 36310 Vigo, Spain

**Keywords:** adsorption, MOFs, microfluidic chip, drug delivery, sodium alginate

## Abstract

Drug delivery systems (DDS) are continuously being explored since humans are facing more numerous complicated diseases than ever before. These systems can preserve the drug’s functionality and improve its efficacy until the drug is delivered to a specific site within the body. One of the least used materials for this purpose are metal—organic frameworks (MOFs). MOFs possess many properties, including their high surface area and the possibility for the addition of functional surface moieties, that make them ideal drug delivery vehicles. Such properties can be further improved by combining different materials (such as metals or ligands) and utilizing various synthesis techniques. In this work, the microfluidic technique is used to synthesize Zeolitic Imidazole Framework-67 (ZIF-67) containing cobalt ions as well as its bimetallic variant with cobalt and zinc as ZnZIF-67 to be subsequently loaded with diclofenac sodium and incorporated into sodium alginate beads for sustained drug delivery. This study shows the utilization of a microfluidic approach to synthesize MOF variants. Furthermore, these MOFs were incorporated into a biopolymer (sodium alginate) to produce a reliable DDS which can perform sustained drug releases for up to 6 days (for 90% of the full amount released), whereas MOFs without the biopolymer showed sudden release within the first day.

## 1. Introduction

The administration and delivery of therapeutic and diagnostics agents to an afflicted site in the body is one of the major challenges in the treatment of various diseases. Conventional methods of drug administration involve various direct administration strategies, such as oral, ocular, transdermal, and intravenous methods of delivering drugs without any provision of a “carrier”. These methods are sometimes ineffective as they suffer various limitations, such as loss of drug function and efficacy, decreased selectivity and transport to the target site, and undesirable effects on other untargeted tissues. Drug delivery systems (DDS) can overcome all these limitations. Drug delivery systems (drug carriers) maintain the physiochemical properties of the drug under the biological environment of the body and can provide a controlled sustained release of drug molecules over a prolonged time [1]. Some of these carriers can also provide secondary effects, such as optical diagnostics and anti-microbial properties. Based on their origin, the drug delivery systems can be categorized as organic, inorganic, and hybrid systems [2,3,4]. Among the inorganic materials used, one such drug delivery system uses high-surface-area materials, such as metal—organic frameworks (MOFs), for drug delivery.

MOFs are high-surface-area materials (usually >1000 m^2^/g) that can adsorb various materials onto their surface. Due to their extensive physical and chemical properties, MOFs are ideal candidates for various applications, including gas adsorption, energy storage, catalysis, wastewater treatment, and biomedical applications [5,6,7]. Their porous structure and their capacity for surface modification make MOFs potential drug carriers [8,9,10,11,12]. Among various known MOFs, Zeolitic Imidazole Framework-67 (ZIF-67) is one of the least employed frameworks in drug delivery [13,14,15]. ZIF-67 is known to have a very high surface area as well as a simple room-temperature synthetic route. Batch synthesis methods, such as room-temperature direct mixing, solvothermal, and hydrothermal methods, are generally used in the preparation of such MOFs, but recently, microfluidic platforms have been employed to synthesize nanoparticles, including materials such as MOFs [16,17,18]. ZIF-67 contains cobalt (Co^2+^) ions, which can have a cytotoxic effect on the cells, but the addition of other metals such as zinc (Zn^2+^), which is biologically relevant, can improve the physical stability of ZIF-67 without compromising the desired qualities. The microfluidic approach is a more controlled method of nanoparticle synthesis when compared to bulk methods [19]. It provides proper control and regulation of the mixing strategies, flow parameters, and flow rates used to control the physiochemical characteristics of the synthesized nanoparticles. Properties such as crystal size, growth rate, surface functions, and synthesis characteristics such as continuous production, scalability, reproducibility, and uniformity can be controlled and varied according to the function of the nanoparticles and the cost–time constraints of the synthesis process [20,21,22]. Further modification, such as encasement in an organic polymer matrix, helps further preserve the carrier and hence the drug while providing a platform for further modification [23,24,25,26,27]. Sodium alginate is a prime candidate for such application as it is non-toxic, anionic, natural, abundant, and highly biodegradable and biocompatible and has a hydrophilic nature and high porosity and mechanical strength.

The present article aims to provide a comprehensive understanding of the microfluidic synthesis of ZIF-67 as well as its bimetallic derivative Zn-ZIF-67, comparing it to the bulk synthesized ZIF-67 in terms of its structure and drug adsorption/loading properties. Further, sodium alginate beads were synthesized, drug-loaded MOFs were incorporated inside the beads, and the drug release profiles were studied. There have been a very limited number of studies regarding the microfluidic synthesis of ZIF-67. We present a novel synthesis of its bimetallic variant for the first time and its combination with sodium alginate for drug delivery applications. This work aims to increase the understanding of such materials and their potential as functional drug delivery agents.

## 2. Materials and Methods

### 2.1. Materials

The polydimethylsiloxane (PDMS) (SYLGARD 184 silicone elastomer and curing agent) used for device fabrication was purchased from Dow Corning Pvt. Ltd., Bangalore, Karnataka, India. The primary metal salt, Cobalt (II) Nitrate hexahydrate (Co(NO_3_)_2_.6H_2_O), and linker, 2-Methylimidazole (2-MIM), were purchased from AVRA Pvt. Ltd., Bangalore, Karnataka, India. The secondary metal salt, Zinc Nitrate hexahydrate (Zn(NO_3_)_2_.6H_2_O), metal salt (linker), Calcium chloride (CaCl_2_), and biopolymer, sodium alginate, were purchased from NICE Chemicals Pvt. Ltd., Bangalore, Karnataka, India. The drug diclofenac sodium was purchased as tablets from CIPLA, and the tablets were crushed into a powdered form. The MTT assay kit (EZcount MTT Cell Assay Kit) was purchased from HiMedia Laboratories Pvt. Ltd., Bangalore, Karnataka, India. Dulbecco’s Modified Eagle Medium (DMEM), Dimethyl Sulfoxide (DMSO), and phosphate-buffered saline (PBS) buffer were purchased from Sigma Aldrich (India) Pvt. Ltd., Bangalore, Karnataka, India. MCF-7 breast cancer cells were acquired from the Department of Biotechnology, JAIN (Deemed-to-be-University), Karnataka, India.

### 2.2. Microfluidic Device Fabrication

**Figure 1:**
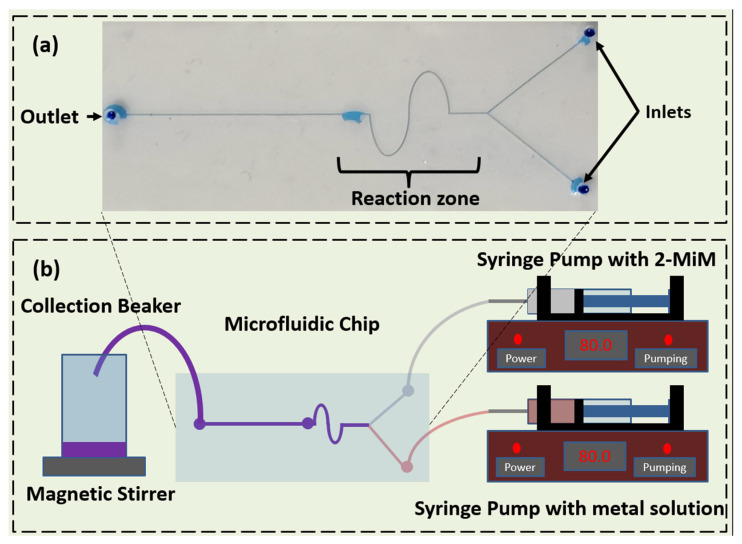
The (**a**) microfluidic chip with the inlets, channels, and reaction zone shown (dyed with methylene blue dye) and (**b**) setup for the synthesis of MOF materials.

The PDMS microfluidic synthesis chip (as shown in Figure 1) was fabricated using the standard photolithographic technique with a channel width and depth of 150 and 100 µm, respectively, with the design based on a microfluidic device for fluoride detection previously reported by our group [28]. There are two inlets and one outlet with 1 mm polytetrafluoroethylene (PTFE) tubings inserted into them. The tubings are connected to their respective syringes (or let into a glass beaker for the outlet tubing). The ‘S’-shaped part of the chip promotes the vigorous mixing of reactants; the cylindrical region (diameter = 2 mm) present after the mixing of the reactants promotes further mixing and might cause larger, heavier-sized particles (>1–2 µm) to settle down due to loss of fluid velocity and also promote the hydrodynamic focussing of homogenous particles towards the outlet.

### 2.3. Synthesis Methods

#### 2.3.1. Synthesis of ZIF-67 and Zn-ZIF-67

Conventional synthesis of ZIF-67 was performed by dissolving 897 mg of Co(NO_3_)_2_.6H_2_O and 1982 mg of 2-MIM in 30 mL of methanol each via sonication. Both solutions were mixed in a beaker and stirred continuously for 24 h. The precipitate was collected, washed with methanol, and dried in a hot air oven overnight. A Y-channel with a serpentine mixing region was used for the microfluidic synthesis of ZIF-67 and Zn-ZIF-67 (MZIF-67 and MZnZIF-67, respectively) (Figure S1). For ZIF-67, 448 mg of Co(NO_3_)_2_.6H_2_O and 991 mg of 2-MIM were dissolved in 30 mL of methanol. Co(NO_3_)_2_.6H_2_O solution was passed through one inlet and 2-MIM solution through the other inlet with a flow rate of 80 µL/min. The product solution was collected from the outlet, washed with methanol, and dried overnight. For Zn-ZIF-67, Co(NO_3_)_2_.6H_2_O salt and zinc nitrate salt were taken in the molar ratio of 1:0.4 (582 mg:238 mg); the salts were dissolved in methanol. Similar to ZIF-67 microfluidic synthesis, salt solution and linker solution were pumped at 80 µL/min (flow rate was chosen based on synthesis time and yields obtained), and the product was collected at the outlet (as shown in Figure 1). The product was washed multiple times with ethanol and dried overnight [16,18,29]. The concentrations of materials were chosen based on the assumption that there should be a minimum ‘build-up’ of material in the channel to prevent its failure, while also considering a reasonable yield of material produced. The flow rate was chosen after conducting a time versus chip failure (blockages, leakages) probability after performing five trials of flow analysis for 30 min at 40, 60, 80, and 100 µL/min. It was observed that a flow rate of 80 µL/min produced the lowest number of chip failures for a decent amount of material produced.

#### 2.3.2. Drug Loading

Diclofenac sodium (DS) was chosen as the model drug. It shows a characteristic peak at around 278 nm under UV–visible spectrophotometry. Various drug concentrations, namely 40, 50, 60, 70, 80, 90, and 100 ppm, were prepared by mixing the drug in de-ionized (DI) water.

#### 2.3.3. MOF–Alginate Bead Synthesis

The bead formation method involves the addition of 5 mg of MOF to 2.5 mL of 90 ppm drug solution followed by agitation for 2 h for adsorption. The resultant solution was mixed with 2.5 mL of 10% (by w.t.) sodium alginate (SA) solution to prepare a 5% SA solution. This solution was pumped at 100 µL/min to produce small-sized drops which were dropped into a linker solution containing 5% CaCl_2_ (by w.t.) (Figure S2). The beads were kept in the linker solution for 6 h for optimal linkage. The beads were then washed with water and dried in an oven overnight. The drying consequently reduced the size and weight of the beads to approximately 1 mm and 1 mg in diameter and weight, respectively, which theoretically should contain 25 µg of MOF per bead.

### 2.4. Characterization

All the materials (ZIF-67, MZIF-67, and MZnZIF-67) were analysed with various analytical techniques to understand their physical and chemical properties. The surface morphology of ZIF-67, MZIF-67, and MZnZIF-67 was determined using the field-emission scanning electron microscopic (FESEM) technique (HITACHI SU-70). Brunauer, Emmett, and Teller (BET) analysis was carried out to determine the multipoint surface area, and a microporous (MP) study was conducted to calculate the total pore volume. An adsorption–desorption study was performed using N2 at liquid nitrogen temperature (−196 °C) on Belsorp-Max (M/s. Microtarc BEL, Osaka, Japan). During the sample analysis process, all the materials were subjected to a degassing process to expel the moisture content present at 100 °C for 2 h. To study the structural features, powder X-ray diffraction (XRD) patterns for the samples were recorded on an Ultima-IV X-ray diffractometer (M/s. Rigaku Corporation, Tokyo, Japan) with Ni-filtered Cu Kα radiation (1 = 1.5406 A°) with a 2θ scan speed of 2 degrees/min and a scan range of 5 to 80 degrees at 40 KV and 30 Ma. Zetasizer Nano ZS—ZEN3600 was used to perform zeta analysis of the samples at 7 pH. X-ray photoelectron spectroscopy (XPS) was used to analyse the chemical states of the elements in the sample using VG multi-lab 2000, which was operated at 3.125 meV using an Al-Kα as the energy source. The MTT assay was carried out using a microplate reader (Molecular Devices SpectraMax ABS Plus, TCL Asset Group Inc. Concord, Canada).

### 2.5. Adsorption Studies

Adsorption studies were performed by changing the two main reaction parameters, such as concentration and time of contact. The concentration studies were performed by adding the respective MOF powders to solutions with different drug concentrations, namely 40, 50, 60, 70, 80, 90, and 100 ppm, followed by shaking for 2 h. After 2 h, the solutions were centrifuged at 3000 rpm to separate the MOF powders and the supernatant was collected. The time studies were performed by adding MOF powders to 90 ppm solutions which were left for adsorption for up to 10, 20, 30, 40, 50, and 60 min, after which the solutions were centrifuged at 3000 rpm to separate the MOF powders and the supernatant was collected. The supernatant was analysed using a UV–visible spectrometer. The drug loading percentage (*C_l_* was calculated using the following equation:

$$C_{l}=\frac{\left(C_{i}-C_{e}\right)}{C_{i}}\times 100$$

where *C_i_* is the initial concentration and *C_e_* is the drug concentration after adsorption.

### 2.6. Drug Release Studies

Drug release studies were performed by the addition of MOF powders and beads to phosphate-buffered saline (PBS, pH-7.4) for a time period of 3, 24, 48, 72, 96, 120, or 144 h, after which the supernatant was collected and analysed using a UV–visible spectrometer. The drug release percentage (*C_r_*) was calculated using the following equation:

$$C_{r}=\frac{C_{e}}{C_{i}}\times 100$$

where *C_i_* is the initial concentration and *C_e_* is the drug concentration after release.

### 2.7. Cytotoxicity Studies

A 48 h MTT assay was conducted with MCF-7 breast cancer cells in a 96-well plate (200 µL). The materials were dispersed (partially dissolved) in a PBS–ethanol mixture with a volumetric ratio of 1:0.05 of PBS to ethanol to enhance the materials’ solubility. A 100 µL solution of an equal population (10,000 cells) of MCF-7 cells in DMEM (with the addition of streptomycin to prevent bacterial growth) was filled in each well, and each plate (one for 24 h and the other for 48 h) was incubated for 24 h. After 24 h, each well was noted, and control populations along with 10 µL of solution with a known concentration of the materials were added to the wells (same volume of blank solution was added to the control wells). The endpoint masses of materials in the wells were 0.003 mg, 0.004 mg, 0.005 mg, 0.006 mg, and 0.007 mg for ZIF-67 and 0.0075 mg, 0.01 mg, 0.0125 mg, 0.015 mg, and 0.0175 mg for MZIF-67 and MZnZIF-67. The different concentrations were taken due to the low solubility/dispersibility of conventional ZIF-67 in PBS. After 24 h and 48 h, a 10 µL solution of MTT reagent (concentration of 5 mg/mL) was added to each well and left for 3 h for the cell–reagent interaction to take place [30]. After three hours, DMSO was added to the wells, the absorbance was measured using a microplate reader at 540 nm, and the OD values were noted. The assay could not be performed for the beads as even after the addition of streptomycin the solution in the wells became turbid and produced improper OD values.

## 3. Results

### 3.1. Characterization

**Figure 2:**
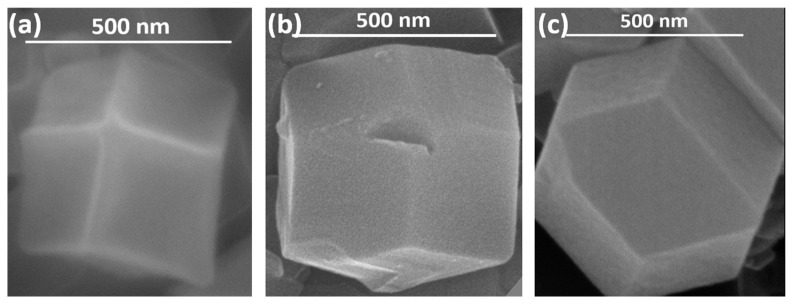
FESEM images of (**a**) ZIF-67, (**b**) MZIF-67, and (**c**) MZnZIF-67.

The FESEM images of the single crystals of the MOF variants can be seen with their respective size bars in Figure 2. The comparison indicates that the crystal sizes of the batch produced and the microfluidically synthesized MOF variants have similar dimensions with a total length of 500 nm (with edge lengths of approximately 250 nm). The images show the crystals to have a similar structure as well as a cubic symmetry, indicating the formation of ZIF-67.

**Figure 3:**
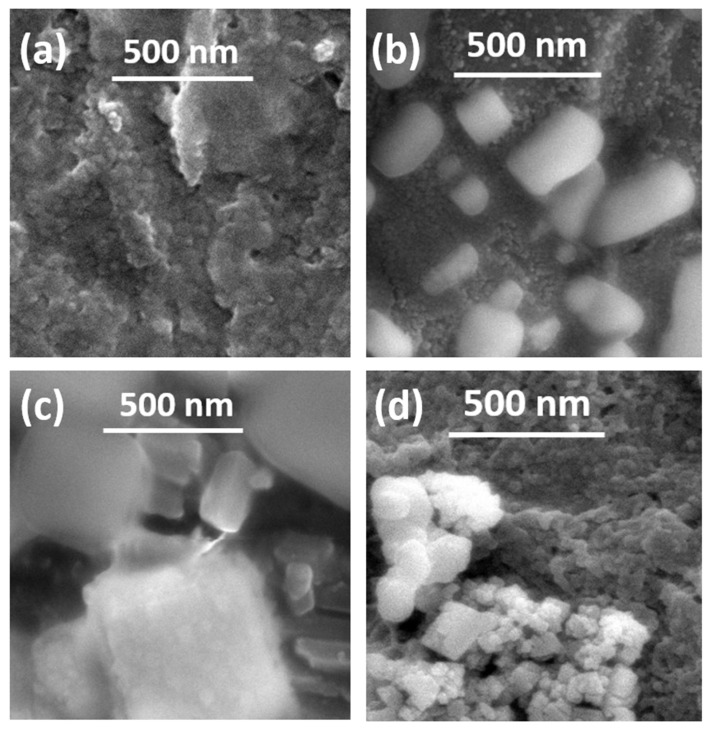
FESEM images of (**a**) ALG, (**b**) ALG_ZIF-67, (**c**) ALG_MZIF-67, and (**d**) ALG_MZnZIF-67.

After the synthesis of MOF-incorporated alginate beads, FESEM images of the beads were obtained, as shown in Figure 3. The alginate cross-sectional surface can be seen in beads devoid of MOF, as shown in Figure 3a, whereas in other images (Figure 3b–d), the MOF crystals (as small clusters) are seen embedded in the alginate macro-structure. The images are meant to show the presence of crystals in the alginate structure, i.e., clusters of smaller crystals are specifically shown (the scale bar is for reference only).

**Figure 4:**
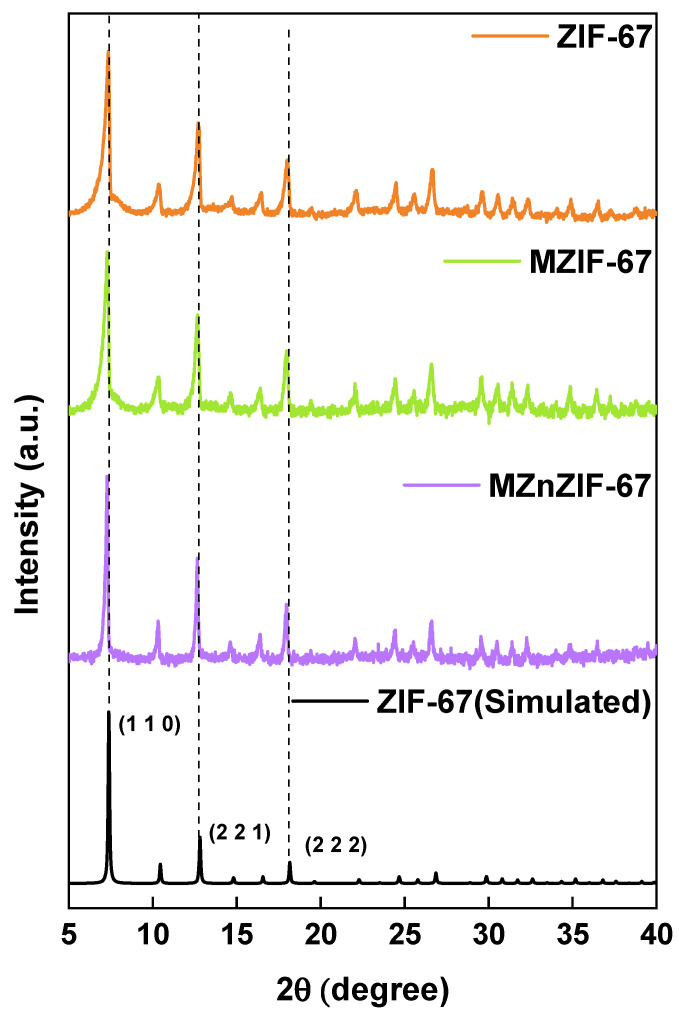
XRD patterns of different MOF materials.

The XRD plot of the MOF variants is shown in Figure 4. It can be observed from the plot that the peaks of the synthesized materials match the simulated ZIF-67 XRD pattern. A comparison of the major peaks ((110), (211), (222)) of all three variants shows that the batch and microfluidically synthesized variants have peaks lying at the same 2θ values. All the major peaks are singular in nature, indicating the absence of bi-phasic or two different materials, especially in the case of MZnZIF-67. The zeta potential was determined to be −12.8 mV, −14.9 mV, and −14.1 mV for ZIF-67, MZIF-67, and MZnZIF-67, respectively.

**Figure 5:**
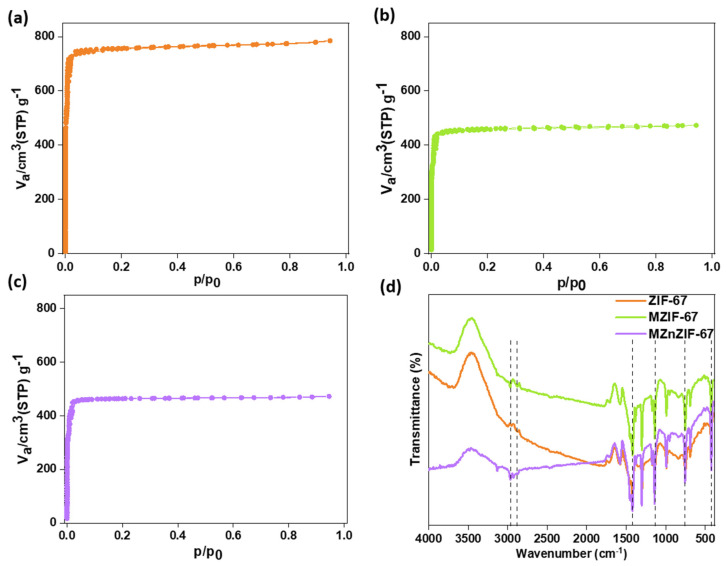
BET plots of (**a**) ZIF-67, (**b**) MZIF-67, and (**c**) MZnZIF-67 and (**d**) FTIR plots for the synthesized materials.

BET isotherms of the materials are plotted and shown in Figure 5. All materials show a Type-I isotherm, indicating that the materials are microporous in nature, owing to their very high surface area. The pore diameters of the materials have similar dimensions, but the overall surface area of batch-synthesized ZIF-67 is higher than that of microfluidically synthesized variants (shown in Table S1). The FTIR spectra of all materials are shown in Figure 5d, with the dotted lines marking the peaks. The peak at 431 cm^−1^ represents the presence of the Co-N bond (and Zn-N in the case of MZnZIF-67), whereas peaks at 760, 1140, and 1438 cm^−1^ indicate the stretching and bending modes of the imidazole ring. The stretching mode of C–H from the aromatic ring and the aliphatic chain in 2-MIM are also described by peaks at 2948 cm^−1^ and 2880 cm^−1^.

**Figure 6:**
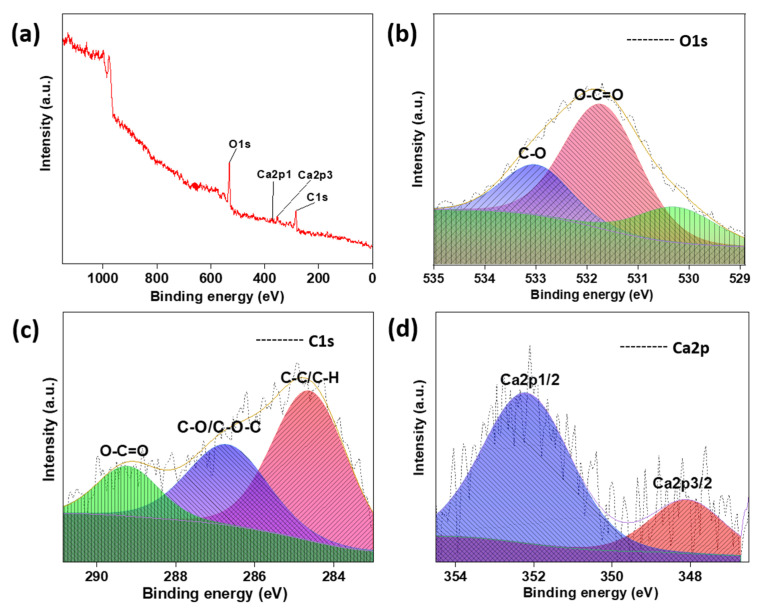
The XPS (**a**) survey spectra of Alg_MZnZIF-67, (**b**) high-resolution spectra of O1s, (**c**) high-resolution spectra of C1s, and (**d**) high-resolution spectra of Ca2p.

X-ray photoelectron spectroscopy (XPS) is a surface analysis technique that describes the elements present as well as their oxidation states and binding information. As can be observed in Figure 6a and Table S2, the elemental composition of the surface of Alg_MZnZIF-67 consists of carbon present in a C1s state with the de-convolution of its peak indicating peaks at 284.65 eV (C–C/C-H), 286.75 eV (C–O/C-O-C), and 289.27 eV (COO-), as well as oxygen in an O1s state with the de-convoluted peaks at 531.73 eV (O-C=O) and 533.05 eV (C-O) [31,32].

### 3.2. Adsorption Studies

#### Concentration and Time Studies

**Figure 7:**
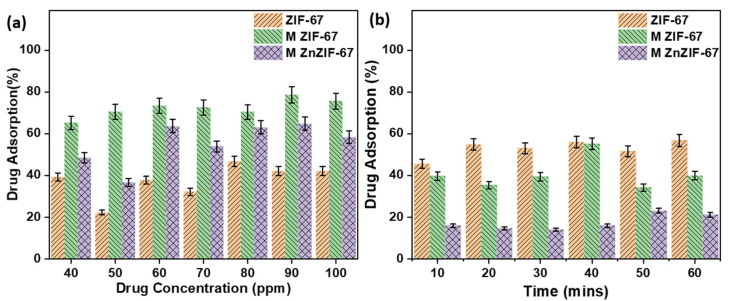
The plot of drug adsorption (%) with respect to (**a**) drug concentration (ppm) and (**b**) time (minutes) for the synthesized MOFs.

Concentration-based adsorption studies are generally performed to observe the maximum adsorption a material (in this case MOFs) can reach over a long period. It can be seen from Figure 7a that the MZIF-67 variant shows the highest drug adsorption across all concentrations of the drug, followed by MZnZIF-67 and ZIF-67. All three variants, MZIF-67, MZnZIF-67, and ZIF-67, reach their highest adsorption capacities of 78%, 63%, and 52%, respectively, at higher concentrations of 80 to 100 ppm (later, 100 ppm was chosen to perform time studies).

### 3.3. Drug Release Studies

**Figure 8:**
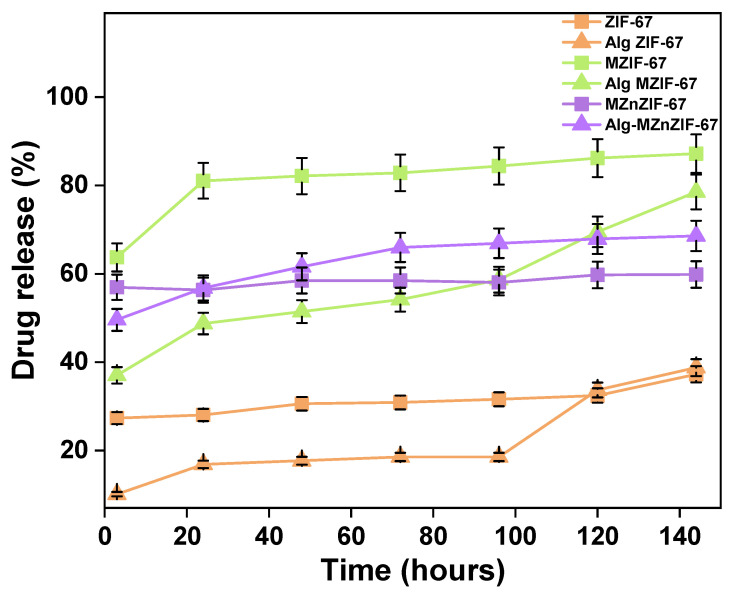
Plot for drug release over time of different MOF and MOF in alginate variants.

The release percentage of the drug in PBS over a period of 3 to 144 h (0 to 6 days) for all MOF and MOF in alginate variants is shown in Figure 8. It can be seen that the powder variants (ZIF-67, MZIF-67, and MZnZIF-67) release most of the adsorbed drug within the first 24 h (1 day), whereas the alginate variants (Alg ZIF-67, Alg MZIF-67, and Alg MZnZIF-67) release their drug content slowly over a period of 144 h (6 days). The drug release of different materials starts at different values because they adsorbed different amounts of the drug, as can be seen from Figure 7a.

### 3.4. Cytotoxicity Studies

**Figure 9:**
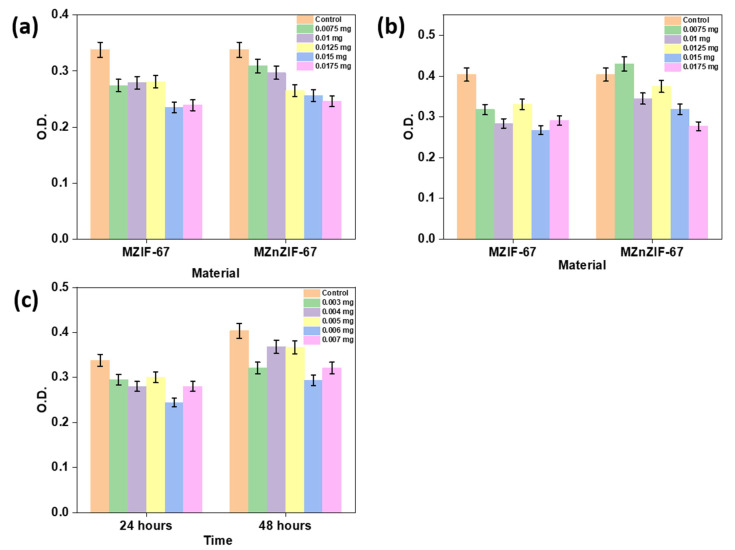
The plot of the OD values with respect to (**a**) materials (MZIF-67 and MZnZIF-67) at different concentrations for 24 h assay, (**b**) materials (MZIF-67 and MZnZIF-67) at different concentration for 48 h assay, and (**c**) ZIF-67 at different concentration for 24 and 48 h assay.

The results of the MTT assays performed on the materials are shown in Figure 9. As can be seen in Figure 9c, the ZIF-67 variant has an erratic OD value trend with comparative cytotoxicity between 24 and 48 h showing a sudden increase from 24 to 48 h. On the other hand, it can be observed from Figure 9a,b that the assays of materials MZIF-67 and MZnZIF-67 show an expected (mostly gradual, non-erratic) trend in OD values, with comparative cytotoxicity between 24 and 48 h showing small increase from 24 to 48 h.

## 4. Discussion

### 4.1. Characterization

The FESEM images (Figure 2) prove that the synthesis of similarly sized crystals of MOFs can be achieved by microfluidic means in a shorter time than by the batch synthesis techniques. The observed size distribution of crystals achieved by both techniques was also comparable, while when embedded in the alginate beads (Figure 3), the distribution of these crystals seems to be in small clusters, owing to the inability of the MOF to be evenly dispersed in the highly viscous alginate (in water) solution. These small clusters are dispersed evenly in the alginate structure, not affecting the overall distribution/dispersion of the MOF crystals in the alginate microstructure. The presence of matching sharper peaks seen in the XRD plots (Figure 4) indicates the formation of highly/purely crystalline expected materials, which in turn dictates that microfluidic processes can reproduce such materials with ease. The decrease in surface areas (surface parameters calculated using the instrument software), as seen from the BET plots (Figure 5), might be due to changes in surface morphology/structure or the introduction of surface moieties due to microfluidic synthesis conditions and reduced synthesis time [33,34]. The high surface area and large pore volume may help in the loading of a large amount of drug molecules, but the same effect may be produced even at a lower surface area along with the presence of certain surface moieties or morphology. This might be supported by the fact that the zeta potential of the microfluidic variants is more negative than that of the bulk variant. The FTIR plots (Figure 5) also confirm the presence of the expected functional groups and thus further confirm the formation of the expected products. The XPS spectra indicate the presence of elements and bonds expected in alginate but the absence of any elements and bonds associated with the MOF embedded in the alginate matrix [35]. It can be gathered from the XPS plots that the metal salt (Ca^2+^ present in Ca2p state) used as a linker is present in the matrix, whereas the metals used for the MOF (Co^2+^, Zn^2+^) are not present, indicating that the MOF is properly embedded into a properly linked matrix. Similarly, Na^2+^ is also not present, which indicates two facts, namely, (i) the alginate matrix has linked properly such that no sodium alginate molecules are present and (ii) there is no leakage of sodium diclofenac from the MOF onto the surface of the beads.

### 4.2. Adsorption Studies and Drug Release Studies

As seen from Figure 7 and Figure 8 and Table S1, three possible reasons can be drawn as to why microfluidic ZIF variants show higher adsorption than the batch-synthesized variant: (a) the stability of the batch-synthesized ZIF-67 in water was observed to be lower than that of the microfluidic variants, which may lead to a decrease in adsorption over time; (b) the faster microfluidic synthesis might lead to the introduction of defects and vacant sites, which might lead to the exposure of functional groups which may increase the adsorption capacity; and (c) a smaller pore size (for microfluidic ZIF-67) suggests that there is a possibility of the drug molecule occupying pores much better than the bigger pores of the batch-synthesized ZIF-67 (as drug molecules may move back and forth from the pore, constantly). The oscillatory nature of the drug adsorption values may be due to the constant adsorption and desorption of the drug molecule from the MOF’s surface due to the processes of diffusion and dissolution [36]. This is also supported by the trend seen in Figure 8, where these MOFs release the majority of the drug molecules almost instantly.

The drug release profiles (Figure 8) of the various materials indicate that the MOF powders (specifically ZIF-67 and MZIF-67) have an erratic, non-uniform release or sudden pre-release where the release of drug molecules may be caused by the breakdown of the structure in the PBS within a short period of time. The MOF-in-alginate beads have a slow overall uniform release over 6 days (and also have a slower release in starting compared to their MOF-only counterparts) due to the preservation of the MOF structure in the alginate matrix and the slow breakdown of alginate in physiological conditions. Among all the MOF-in-alginate variants, the Alg-MZnZIF-67 variant shows the best uniform release, which may be due to the stability of MZnZIF-67 in PBS being higher than that of other variants due to the incorporation of zinc along with cobalt in the MOF structure [29].

### 4.3. Cytotoxicity Studies

The ZIF-67 has a highly toxic effect on the cells even at very low concentrations compared to the microfluidically synthesized materials, and the erratic OD values may support its unstable nature in aqueous solutions as it might be rapidly degrading in the solution, thus making it unsuitable as a drug carrier. The assays of materials MZIF-67 and MZnZIF-67 show an expected (mostly gradual, non-erratic) trend in OD values, which may support that the materials are stable in aqueous medium for at least up to 48 h. Among these two materials, MZnZIF-67 shows a much more stable trend of increasing cytotoxic effect with an increase in material amount, with the MZIF-67 having lower OD values than those of MZnZIF-67 for the same corresponding amount of material.

## 5. Conclusions

It can be gathered from the aforementioned data that MOFs can be used for drug delivery purposes after appropriate modifications. The results show that the microfluidic method produced homogenous MOF particles similar to batch/bulk processes in a short amount of time, as well as improved the particles’ adsorption properties. All three synthesized variants follow Freundlich isotherm, which indicates multi-layered adsorption, and follow the second-order kinetics as well. The microfluidically synthesized variants show a higher adsorption capacity as the synthesis may have added or left some positive functional groups on the surface of the MOF particles (which might have also led to a decrease in surface area; this requires further investigation) which is supported by the fact that many MOF structures adsorb organic molecules via electronic interactions and hydrogen bonding as well as π-π stacking, i.e., diclofenac molecules may interact with positive surface moieties to be adsorbed. Even though such materials are unsuitable for drug delivery purposes due to the presence of ‘toxic’ ions such as cobalt and being unstable under physiological conditions, they can be further improved by combining other metals such as zinc to greatly improve their stability and further reduce toxicity (as can be seen by the MTT assay studies) (as shown in Figure S3). As the studies were performed on cancer cells and showed some cytotoxic effect towards these cells, this also supports the possibility of the use of such toxicity in a favourable way, as if the particle can be inserted or directed towards an affected area, the release of toxic ions can help kill the invading/infected moiety (bacteria, fungus, etc.) as well as destroy cancerous tissue while delivering the relevant medication. Subsequently, the incorporation of such materials in a biopolymer (sodium alginate) matrix helps further protect the delivery agent (MOFs) (slowing down the framework’s (MOF’s) breakdown) and the drug, thus making them biocompatible, safe, and easy to transport. These beads slowly release the drug over a long time of up to 6 days. MOFs possess many physiochemical properties that make them ideal drug delivery agents, but their qualities can be further improved by the utilization of different synthesis techniques and combinations of different metals in the structure as well as the incorporation of such materials in a biopolymer, as shown in this work.

## Data Availability

The data presented in this study are available on request from the corresponding author.

## Supplementary Materials

The following are available online at https://www.mdpi.com/article/10.3390/bios13070737/s1, Figure S1. Setup for microfluidic MOF synthesis; Figure S2. Schematic of setup for MOF-in-alginate bead synthesis; Figure S3. Stability of microfluidically synthesized MOF variants in PBS over a period of 5 days; Table S1. BET isotherm parameters of different MOFs; Table S2. The binding energies and elemental composition from the XPS of Alg_MZnZIF-67.
