# Supplementary material for: Microfluidic-Assisted Synthesis of Metal—Organic Framework —Alginate Micro-Particles for Sustained Drug Delivery
Source: Biosensors (Basel). 2023 Jul 17;13(7):737. doi: 10.3390/bios13070737 (PMC10377693; doi:10.3390/bios13070737)
Supplement: Supplementary file 1 [file biosensors-13-00737-s001.zip › biosensors-2383813-supplementary.pdf]

biosensors

Supplementary Materials

Microfluidic-Assisted Synthesis of Metal—Organic Framework—Alginate Micro-Particles for Sustained Drug Delivery

Akhilesh Bendre [1], Vinayak Hegde [1], Kanalli V. Ajeya [2], Subrahmanya Thagare Manjunatha [3], Derangula Somasekhara [4], Varalakshmi K. Nadumane [4], Krishna Kant [5,*], Ho-Young Jung [2,*] Wei-Song Hung [3,*] and Mahaveer D. Kurkuri [1,*]

1 Centre for Research in Functional Materials (CRFM), JAIN (Deemed-to-be University), Jain Global Campus, Bengaluru 562112, Karnataka, India; akhilesh.bendre@jainuniversity.ac.in (A.B.); vinayakhegde2020@gmail.com (V.H.)
2 Department of Environment and Energy Engineering, Chonnam National University, 77 Yongbong-ro, Buk-gu, Gwangju 61186, Republic of Korea; ajeyhegde94@gmail.com
3 Advanced Membrane Materials Research Center, Graduate Institute of Applied Science and Technology, National Taiwan University of Science and Technology, Taipei 10607, Taiwan; mssubrahmanya@gmail.com
4 Department of Biotechnology, JAIN (Deemed-to-be-University), School of Sciences, JC Road, 34, 1st Cross Road, Sudharna Nagar, Bengaluru 560027, Karnataka, India; d.somasekhara@jainuniversity.ac.in (D.S.); kn.varalakshmi@jainuniversity.ac.in (V.K.N.)
5 Biomedical Research Center (CINBIO), University of Vigo, 36310 Vigo, Spain
* Correspondence: krishna.kant@uvigo.es (K.K.); jungho@chonnam.ac.kr (H.-Y.J.); wshung@mail.ntust.edu.tw (W.-S.H.); mahaveer.kurkuri@jainuniversity.ac.in (M.D.K.)

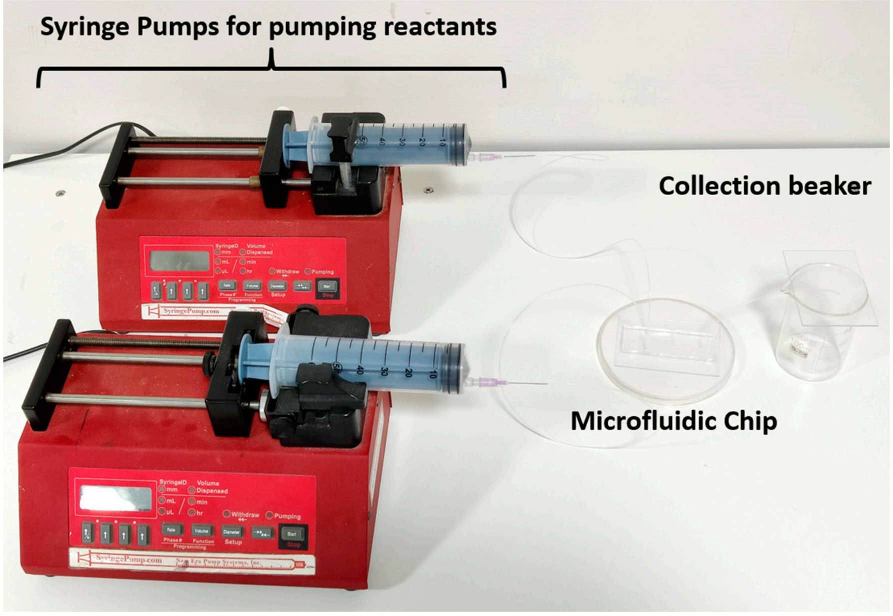

Figure S1. Setup for microfluidic MOF synthesis.

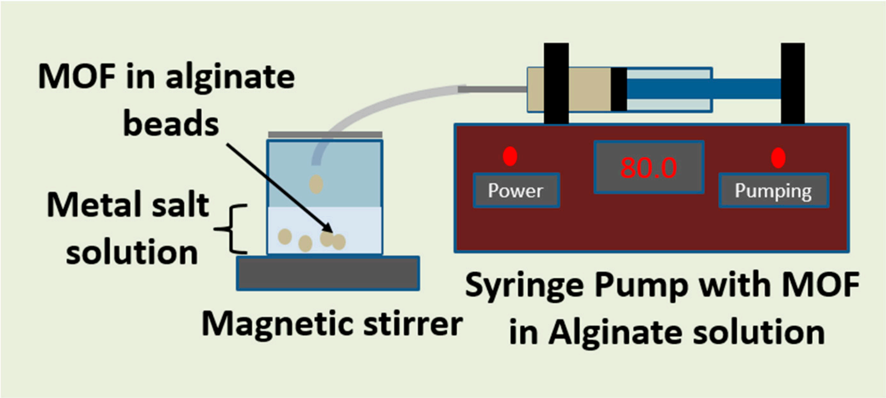

Figure S2. Schematic of setup for MOF in Alginate bead synthesis.

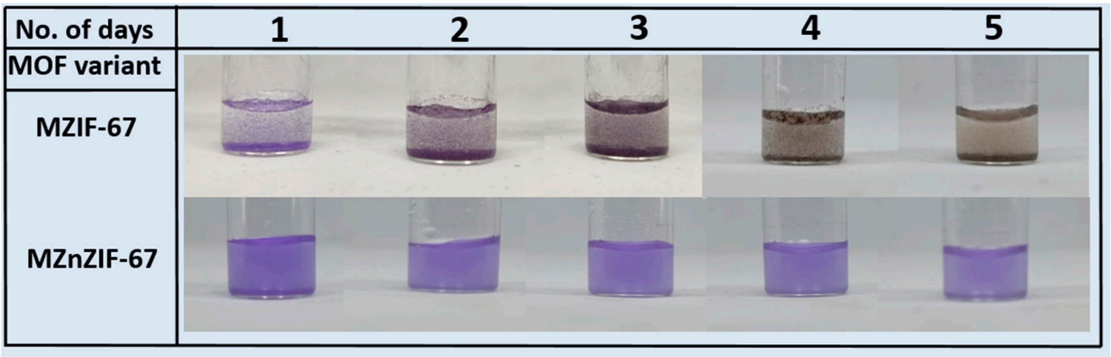

Figure S3. Stability of microfluidic synthesized MOF variants in PBS over a period of 5 days.

Table S1. BET isotherm parameters of different MOFs.

Type of MOF/Adsorbent	$a_{s,BET}$ (m^2g^{-1})	Mean pore diameter (nm)	Total pore volume (cm^3g^{-1})
**ZIF-67**	3001.3	1.6177	1.2138
**MZIF-67**	1823.4	1.6057	0.7319
**MZnZIF-67**	1864.3	1.5674	0.7305

Table S2. The binding energies and elemental composition from the XPS of Alg_MZnZIF-67

Name	Binding Energy (eV)	Atomic %
**C1s**	284.6	48.58
**O1s**	531.07	45.96
**Ca2p3**	351.03	1.58
**Ca2p1**	354.4	1.99
